# Dignity and Human Rights—Survey Findings on Undergraduate Nursing Students' Conceptualisation and Operationalisation of Dignity

**DOI:** 10.1002/nop2.70194

**Published:** 2025-04-01

**Authors:** Sheila Douglas, Leah Macaden, Kevin Muirhead, Elaine Webster, Liz Ellis

**Affiliations:** ^1^ Nursing and Health Sciences University of Dundee Dundee UK; ^2^ Nursing Studies, School of Health in Social Science University of Edinburgh Edinburgh UK; ^3^ Hillcrest House Carers Scotland Nairn UK; ^4^ The Law School University of Strathclyde Glasgow UK; ^5^ University of South Wales Pontypridd UK

**Keywords:** co‐design, dignity, human rights, nurse education, students, undergraduate nursing

## Abstract

**Aim:**

The aim of the study was to explore how students conceptualise and operationalise dignity with confidence in practice through a human rights lens.

**Design:**

A quantitative study using an online survey questionnaire.

**Methods:**

Data were collected using an online survey with 33 questions in three parts: students' conceptualisation of dignity, understanding of human rights and human rights law; students' operationalisation of dignity using a case study designed for this purpose [a fictional character named John]; lastly, students' preferred approaches to dignity education.

**Results:**

Survey findings revealed students' ambiguity or lack of agreement around dignity being associated with human rights and person‐centred care. There was a sense of students feeling disempowered or lacking confidence in responding to dignity breaches in care, whilst some participants felt equipped to challenge this by most usually referring to clinical staff such as mentors and charge nurses due to the hierarchy in nursing systems within clinical contexts.

**Conclusion:**

Informed by the findings from this survey, the research team has developed DigniSpace (2024), the first online interactive space for Dignity Education co‐produced with students focusing on the concept of dignity (through a consideration of human rights) that has been designed to help students learn more about the concept and to confidently promote and advocate dignity in practice. This is the first such resource to empower students to interrogate the concept of Dignity using the human rights lens and become change agents to promote and advocate dignity in care as a fundamental human right in any practice context.

**Implications for Nursing:**

Findings and outputs from this research have used the context of nursing education as a critical opportunity by placing students at the heart of developing DigniSpace (2024) to support the sustainable development of a culture of confidence to provide person‐centred care embedded with dignity.

**Patient or Public Contributions:**

Findings from this first phase of the study were presented to our project advisory group that included experts with lived experience and their family care partners, who then participated in the co‐design workshops in the second phase of the study to develop DigniSpace—a key output from this project.

## 1 Introduction and Background

Dignity—as practiced by practitioners in health and social care environments and as experienced by people who access health and social care services—fundamentally underpins health and social care law and policy across the United Kingdom. There is a wide evidence base on concerns over dignity in practice. Much is focused on specific client groups such as older adults and those experiencing dementia, but wider concerns are also reported (Digby et al. 2017; Boddington and Featherstone 2018; Scerri et al. 2020).

### 1.1 Dignity in Nursing Education

The literature on educational strategies to promote dignity as a concept and dignity in practice for students is sparse (Kyle et al. 2017). Papers capture a dual focus: those that seek to facilitate learning for students to deliver dignity in practice, and those that seek to protect students from challenging clinical learning environments and experiences that impact upon their own dignity. Several authors support the possibility and importance of facilitating learning about how to deliver dignity in care (Matiti 2015; Papastavrou et al. 2016); others provide short reports of teaching strategies that have been implemented (Goodman et al. 2018). Kyle et al. (2017) advocate the integration of experiential and experimental educational approaches into undergraduate nursing curricula. Stikholmen (2022a) found that participants revealed learning through the recognition of their own vulnerability, both in social settings and in their education, often using terminology such as pride or shame to exemplify their vulnerability. Vulnerability is an enduring theme in the undergraduate nursing literature (Gallagher et al. 2017; Papastavrou et al. 2016; Stikholmen et al. 2022b; Tehranineshat and Torabizadeh 2021) and it has been linked to burnout and attrition (Levett‐Jones et al. 2009; Murphy et al. 2009; Bickhoff et al. 2016). Kyle et al. (2017) significantly note that ‘guarding’ is necessary to protect students against sustained negative exposures which could lead to undergraduate nursing students ‘unlearning’ dignity within practice cultures.

Jacobson's definition of dignity underpins this study entitled ‘Dignity Engagement Space for Nurse Education using a Human Rights based Approach (DESNEHRA). Jacobson's definition distinguishes between fundamental human dignity and social dignity:

Human dignity is the abstract, universal value that belongs to human beings simply by virtue of being human. As a principle, it admits of no quantity and cannot be created or destroyed. Social dignity is generated in action and interaction. It may be divided into two types: dignity‐of‐self and dignity‐in‐relation. (p. 17)

### 1.2 Human Rights in Nursing Education

The Nursing and Midwifery Council Code of professional conduct (The Code, NMC 2018) portrays a professional landscape of respecting and upholding human rights through the imperative of prioritising people. Human rights and dignity as terms or concepts are not specifically used. Human rights are the focus of a limited section of the nursing literature, while dignity appears extensively (Gallagher 2004). Haddock (1996), in an early concept analysis of dignity, includes human rights as the basis for individual/personal values in the delivery of nursing care. More recently, Hopia and Lottes (2017) evaluate human rights‐based approaches in post‐qualifying nursing education. Exploring the meaning of dignity with master's level nursing students in Finland, they report that studying human rights successfully raised awareness of human rights in patient care and that this new knowledge enabled nurses to better support patients in vulnerable situations. The present study was informed by our earlier pilot research drawing on human rights expertise (Kyle et al. 2017; Macaden et al. 2017; Munoz et al. 2017), but the most recent work foregrounded the human rights framework. So, whilst dignity in nurse education is more usually founded on ethical principles (Gastmans 2012), the project reported here instead adopted a human rights‐based framework which will now be explored.

## 2 The Study

### 2.1 Aim

The aim of the ‘Dignity Engagement Space for Nurse Education using a Human Rights Approach’ (DESNEHRA) study was to explore how students conceptualise and operationalise dignity with confidence in practice, through a human rights lens. It intended to capture students' views prior to the following planned phases of co‐design workshops and the subsequent development of ‘DigniSpace’—an online resource for self‐directed reflective learning that enables students to interrogate the concept of Dignity using a human based approach (Human Rights‐based Approach to Dignity in Care (DIGNISPACE) | Coursera).

### 2.2 Objectives

1. To explore students' understanding and conceptualisation of dignity using a human rights‐based approach;
2. To explore students' confidence in operationalising dignity in practice.

## 3 Methods

### 3.1 Design

This was a quantitative study using an online survey launched via Jisc Survey (JISC 2021). The interdisciplinary research team included expertise in the fields of pedagogy and nursing education, human rights law, participatory research, co‐production, instructional design and educational technology from three Scottish universities.

### 3.2 Sampling

The project aimed to recruit student nurses or students studying on the Higher National Certificate [HNC] courses with potential health/social care futures from two Scottish universities using **convenience sampling**. The sampling frame included participants studying for a number of educational awards, intended to capture the breadth of educational preparation for nursing within the collaborating partners, that is, BSc Nursing; HNC Care and Administration Programme; BA Health and Social Studies; BSc Nursing (Honours); MSc Nursing. There was no sample size calculation used since the intention was to recruit students on the programmes listed above (*n* = 300) using convenience sampling. However, survey respondents representing health and social care education from university 1 were **122** (88.2%) and university 2 were **14** (10.3%), which resulted in a 45.3% response rate overall. Survey respondents largely represented studying Adult Nursing: **111** (81.6%) followed by Mental Health Nursing: **22** (16.2%) (Table 1) which also reflects national trends with field‐specific nursing studies. The survey remained open following ethics approval from July 2019 to January 2020 for data collection.

**TABLE 1:** Demographic data.

University	Number	%
University 1	122	89.7
University 2	14	10.3
**Campus**		
*University 1*		
Campus A	105	77.2
Campus B	1	0.7
Campus C	14	10.3
*University 2*		
Campus A	4	2.9
Campus B	10	7.4
NA^a^	1	0.7
NR	1	0.7
**Course**		
MSc Nursing	3	2.2
MA Health and Well‐being	1	0.7
BSc Nursing	114	83.8
HNC Care and Administrative Programme	8	5.9
Access to nursing	5	3.7
NA^a^	1	0.7
NR	4	2.9
**Year of study**		
First	89	65.4
Second	39	28.7
Third	7	5.1
NA^a^	1	0.7
**Branch of study**		
Adult Nursing	111	81.6
Mental Health Nursing	22	16.2
NA^a^	1	0.7
Other^b^	2	1.5
**Previous experience as health care assistant**		
Yes	84	61.8
No	52	38.2
**Previous experience in practice placement**		
Yes	74	54.4
No	59	43.4
NA	3	2.2

Abbreviations: NA, not applicable; NR, nonresponse.

^a^ NHS employee.

^b^ One participant had experience in all branches, and another had experience as a lecturer.

### 3.3 Recruitment

A PowerPoint presentation on the project was delivered to all students at the two participating universities. Following the presentation, a link to the online survey was made available via the universities' Virtual Learning Environments [VLE] along with an announcement inviting students who were interested to complete the survey [opt in] compliant with the General Data Protection Regulation [GDPR] requirements. Additionally, students were also required to read the participant information sheet [PIS] and complete an online consent form to be able to access the survey. Students who responded to the survey were also invited to participate in co‐production workshops using a separate link to indicate their consent to opt in for the next stage of the project to de‐link the survey participant with the data and ensure the anonymity of survey participants.

### 3.4 Data Collection

The survey included 33 questions in three parts: students' conceptualisation of dignity, understanding of human rights and human rights law; students' operationalisation of dignity using a case study designed for this purpose [a fictional character named John]; lastly, students' preferred approaches to dignity education. Content validity of the questionnaire was established with two experts in nurse education and an expert in human rights law. Reliability testing for the questionnaire wasn't undertaken. The survey remained open.

The initial part of the survey acted as a baseline and informed the learning outcomes and indicative content of DigniSpace (2024).

A case study approach in part 2 of the survey was adopted to facilitate students' thinking and operationalisation of dignity in practice. An exemplar case study entitled ‘John’ (Box 1) was developed by the research team for the students to use as a hook, primarily to report on how they would reflect upon operationalisation of dignity in John's care.

BOX 1 **Case study: ‘John’.**

John is a 70 year old gentleman who lives in rural Scotland. He has been married to Eilidh for the past 40 years. They have two daughters Katie and Carol and a son Rob who are all married and have their own families but live in Scotland; John is a retired teacher, whose hobbies are art, music and gardening. He taught Maths and Physics at school and was a very popular teacher. John loves doing crosswords and playing Scrabble with Eilidh in the evenings at home. They are both actively involved with their local community including their village church.

John was recently diagnosed with Alzheimer's Disease but is very independent and has requested that he would like to continue to be as independent as possible. He goes through his day as he always did, although now Eilidh sometimes has to assist with a task, help with finding the right word or give a friendly reminder. She has a Lasting Power of Attorney for John but continues to include John in decisions, including treatments, future care and finances.

Being an avid gardener John maintained an immaculate garden with many varieties of plants, beautiful shrubs and trees. Having forgotten to wear his glasses whilst working in the garden, John missed a step and had a nasty fall that resulted in a hip fracture. Following a hip replacement, John is admitted to an orthopaedic ward.

The final part of the questionnaire provided the opportunity for students to comment on preferred learning and teaching strategies that would inform the next phase of the project, that of co‐production/co‐design workshops to develop a proposed online learning space for dignity education. This will be presented in a separate paper. Participants' responses to the questionnaire were predominantly via a five‐point Likert scale with space for free text responses as appropriate.

### 3.5 Data Analysis

The survey data was analysed using descriptive statistics in SPSS Version 26 as presented below.

### 3.6 Ethical Consideration

The project received ethics approval from University Research Ethics Committee: Ref No: OLETHSHE1032 dated 30th May 2019.

## 4 Results

This paper reports on the findings regarding students' conceptualisation and operationalisation of dignity in John's care.

### 4.1 Subjects

A total of 136 students completed the online survey between June 2019 and January 2020. Participants' demographic data is displayed in Table 1.

A majority of participants [61.8%] had previous work experience as health care assistants. Students reported clinical placements in a range of clinical settings that is, acute care (44/38.9%); Community Hospital (40/35.4%); District Nursing (27/23.9%); Care Homes (13/11.5%); Care of older adults (25/22.1%); Mental Health Services (30/26.5%);

### 4.2 Students' Knowledge and Understanding of Dignity

Students were given a free text option to state, in **ONE** word, what dignity meant to them. The five most frequently used words (Table 2) give insight into participants' broad definitions and understandings of the concept of dignity, which was overwhelmingly associated with ‘respect.’

**TABLE 2:** Top five words to chosen to describe the meaning of dignity.

	Word	Count
1	Respect	89
2	Privacy	8
3	Worth	5
4	Choice	3
5	Everything	2

Students' conceptualisations of dignity were further measured by Likert‐based questions that sought their level of agreement with several statements about providing dignity in care. These capture the complexity of the concept of dignity as students attempted to analyse and unpack this complex concept.

Results demonstrated students beginning to consider the gap between theory and practice, and between personal values and practice contexts. These dilemmas became increasingly evident as they responded to the triggers in the case study.

**FIGURE 1:**
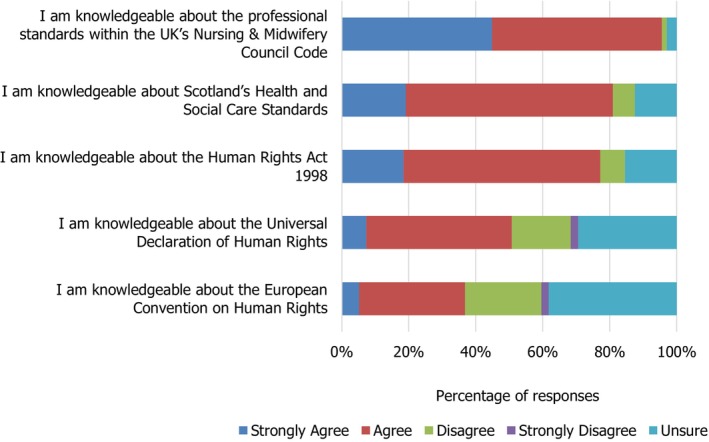
Knowledge of human rights standards and legislation.

As seen in Figure 1, a vast majority of participants strongly agreed/agreed that they were knowledgeable about the NMC Standards [> 95%] and NHS Scotland Health & Social Care Standards [80%] in comparison to the Universal Declaration of Human Rights [50%]. However, it was interesting to note that in relation to responsibility for upholding the human rights of patients, participants agreed/strongly agreed that all nurses have a legal duty to ensure respect for patients' human rights [97.06%]; senior nurses had a legal duty to ensure respect for patients' human rights [95.6%] and Health Service managers had a legal duty to ensure respect for patients' human rights [97.1%]. Further, a more detailed presentation and discussion of the human rights aspects of the study will be reported in a separate paper.

After asking participants to give their views on the meaning of dignity, the relationship between dignity and respect, and abstract associations between dignity and practice contexts, John's case study (See Box 1) was used to explore how students thought about the operationalisation of dignity with the following question:

You are the student nurse assigned to care for John, along with your mentor. “What does dignity mean to you in the following domains of John's care whilst he is in hospital”?

Six key domains were identified for John's care [personal care, food and nutrition needs, communication, meaningful engagement with him, pain and his end of life care] that students would routinely encounter in their everyday practice. These are based upon the Person‐Centred framework adopted by The Health Foundation (2014).

**FIGURE 2:**
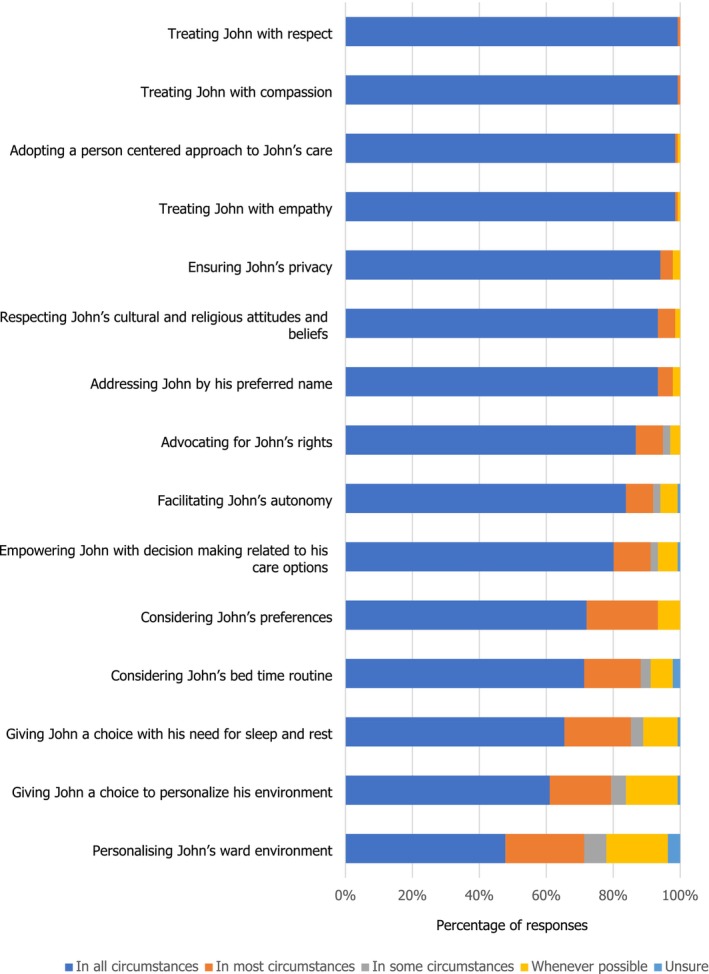
Domains of John's care: personal care.

Most students [> 95%] agreed that caring for John with dignity was inclusive of treating John with empathy, compassion and respect, adopting a person‐centred approach in all circumstances. At the same time, when it came to operationalising dignity with a person‐centred approach [i.e., considering John's preferences with bed time routine, sleep or personalising his environment], only 60%–65% (Figure 2) students interpreted that considering choices/preferences, advocating for John's rights and facilitating his autonomy were required to provide his care with dignity in all circumstances.

**FIGURE 3:**
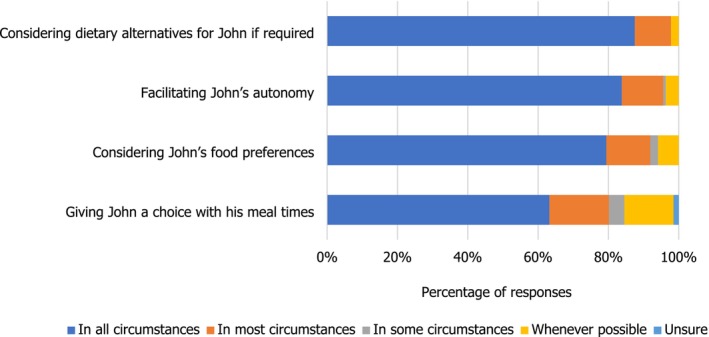
Domains of John's care: food and nutrition.

Similarly, when it came to John's food and nutrition (Figure 3), giving a choice with mealtimes in all circumstances was interpreted as respecting John's dignity only by just over 60% of the students.

**FIGURE 4:**
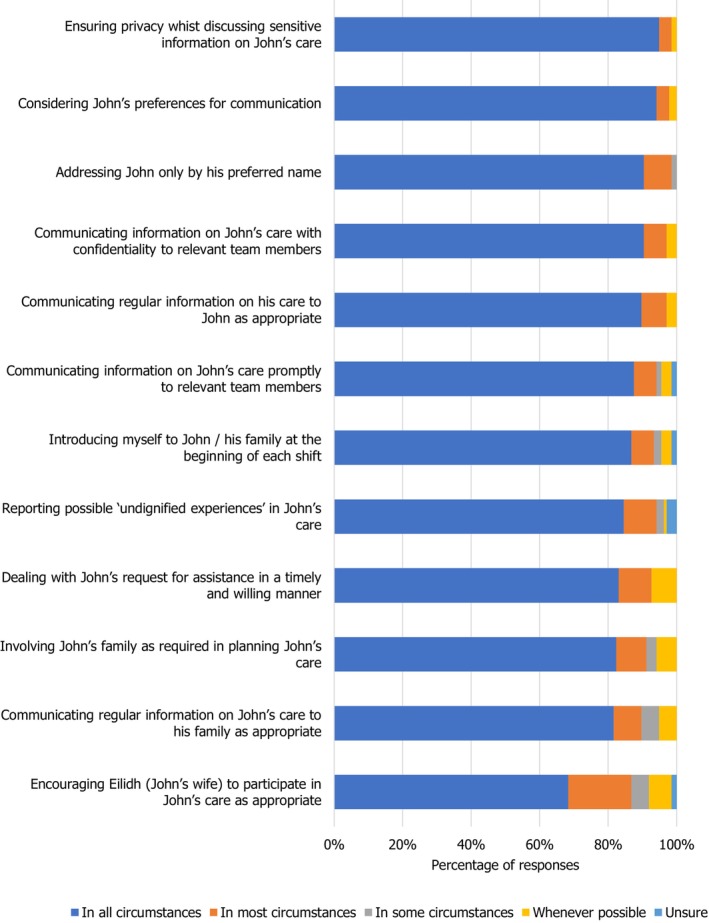
Domains of John's care: communication.

15% of the students (Figure 4) interpreted respecting John's dignity meant: dealing with John's request for assistance in a timely and willing manner and reporting possible ‘undignified experiences’ in John's care in most/some circumstances or whenever possible, as opposed to in all circumstances.

**FIGURE 5:**
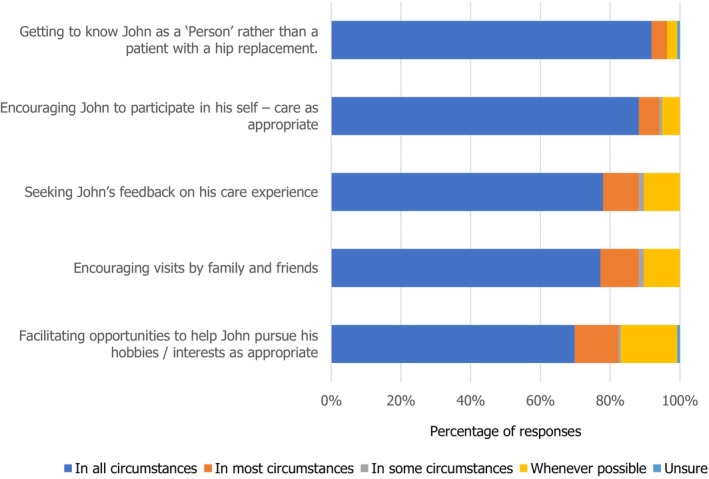
Domains of John's care: meaningful engagement.

Most students interpreted that supporting John with various aspects of meaningful engagement in all circumstances was essential to respecting his dignity (Figure 5). But only 70% of the participants attributed to supporting John to meaningfully engage with hobbies and interests in all circumstances as respecting his dignity.

**FIGURE 6:**
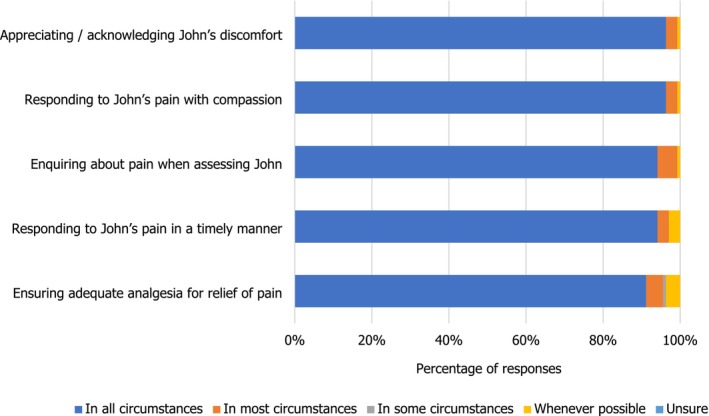
Domains of John's care: pain.

Nearly 95% of the students interpreted a timely and compassionate approach to both assessing and managing John's pain in all circumstances as respecting John's dignity in this domain of his care (Figure 6).

**FIGURE 7:**
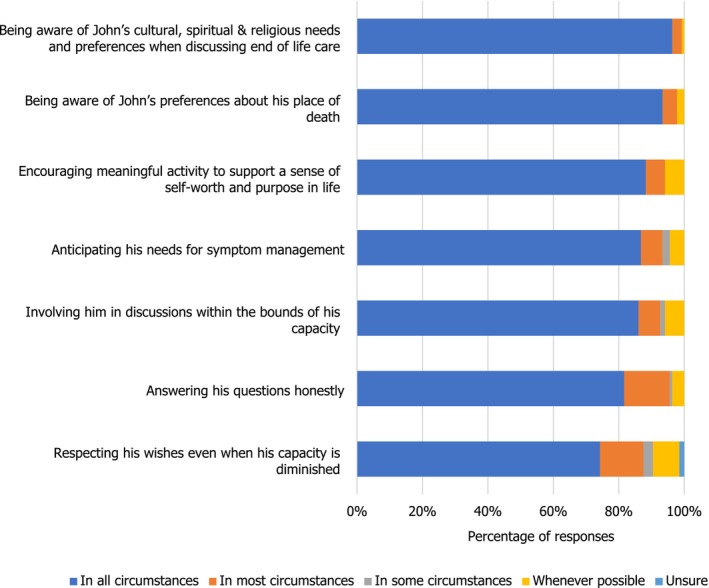
Domains of John's care: end of life care.

Most students interpreted that awareness around John's cultural and religious beliefs, preferences around his place of death and involving him in discussions if he had the capacity in all circumstances were indicative of respecting John's dignity (Figure 7).

Practising dignity in care also requires challenging other professionals when there are ‘dignity breaches’ in care. Free text responses from the questionnaire were invited to capture the ways in which participants responded to undignified care. The researchers were interested in measuring an aspect of dignity that went beyond practising with dignity, to speaking‐up when undignified practice was witnessed as part of how students operationalised their understanding of dignity in practice not just in their own practice but within their practice environment. Participants reported a variety of strategies. These included speaking to the colleague involved and/or challenging the behaviour, discussing the episode with a mentor or senior member of staff, taking immediate action to preserve the dignity of the patient or taking no/limited action. Representative participant quotes are provided to give examples of the main responses to undignified care (Table 3).

**TABLE 3:** Responding to dignity breaches in practice.

Response	Participant Quote/s	Participant ID
Speak to the colleague involved and/or challenge the behaviour	*Ask colleague why they don't feel the need to treat this patient with the level of dignity they are entitled to & discuss other ways in which we could go about his/her care in a more dignified manner*	481765‐481756‐51929672
	*I would try to intervene in a nice way*, e.g., *asking if I can help, or saying to the nurse: “I can see that you are tired, would you like me to take over?”. Later on, I would talk to the nurse in question and ask why she/he was acting this way. Sometimes* e.g., *people come across as being ‘short’ without realising that*, e.g., *if they are tired or overworked*	481765‐481756‐52461755
Discuss the episode with a mentor or senior member of staff	*Report to my mentor or another senior member of staff. If I feel comfortable with the charge nurse, I would confide in her*	481765‐481756‐52498026
	*If a patient was being treated without respect, I would take a pause and approach the ward manager and advise them of the situation*	481765‐481756‐60441401
Take immediate action to preserve the dignity of the patient	*Introduce myself into the situation and approach John with the right attitude, and in a person‐centred manner*	481765‐481756‐51632049
	*If it was something about the environment, I would try to change it immediately*, i.e., *closing a curtain/door if personal discussions are being heard by others*	481765‐481756‐60441401
Take no immediate action	*Probably very little, it is very difficult as a junior member of staff to question a senior members of staff's care approach*	481765‐481756‐48281601
	*I wouldn't do anything immediately*	481765‐481756‐51632209

Participants also suggested that witnessing undignified care could result in enhanced insight and strengthen their resolve and personal commitment to provide dignity in care:

It just encourages me to work harder and maintain an excellent standard of care based on respect and dignity. When I see the practices of others that I feel are lacking, or inappropriate, I feel like it fuels my drive to become better, and influence others to do the same. 481765‐481756‐52498026

Ongoing dialogue and support from senior staff were important for long term, lasting improvements through greater attention to care behaviours and open discussions:

I would talk to my mentor and discuss the appropriateness of the behaviour confidentially to ensure I had an objective understanding of the situation. 481765‐481756‐49394547

Open conversation with others and observe any changes that need to be made. 481765‐481756‐51629362

I would inform the line manager to provide more training on how to respect individual's dignity. 481765‐481756‐50112234

Personal and peer reflections were also considered to be important:

I would reflect on my own practice and also gain some feedback from colleagues and patients as to how my practice is perceived by others so that I could identify any areas for improvement and assess the best way to achieve this. 481765‐481756‐52598281

The findings demonstrated that participants had a strong commitment to improve practice and skills:

I would ensure that my own practice was clearly person centred and based on ensuring the dignity of the patient. 481765‐481756‐50380458

Participants were motivated to become positive role models and demonstrated awareness of how to engage with relevant policy in the event of concerns:

I would consult my mentor and personal tutor and I would also look into the whistleblowing policy. 481765‐481756‐50078937

Several participants also appeared to have the confidence required to report episodes of undignified care:

I would absolutely be reporting to a senior member of staff, after all, you are your patient's advocate and you have to speak up on their behalf. 481765‐481756‐52122579

I would always report behaviour that I thought was not up to standard or practice. 481765‐481756‐50080319

However, other participants did not feel confident or comfortable in reporting other staff, particularly senior staff:

I'd report the behaviour but would be worried about the be consequences of working with the person. 481765‐481756‐51630977

I would feel like it should be flagged up but being a student might not be my place. 481765‐481756‐51631461

Similarly, some participants were reluctant to challenge senior colleagues directly after witnessing undignified care behaviours:

I would like to feel confident enough to address the situation first hand but do not feel that I am in a position to do so and do not want to be confrontational. 481765‐481756‐49951826

I would like to address the person in question but do not feel I would have the experience to do so alone without support of a more senior member of staff. 481765‐481756‐49394547

In summary, participants could state with some confidence what they perceived the role of the nurse to be in relation to the concept of dignity and how it is operationalised in care, both by peers, colleagues and by themselves. This confidence fluctuated in response to personal moral stances and status as a professional learner.

## 5 Discussion

Several areas of concern arose from the data analysis. One of the stated objectives of the study was: ‘To explore students’ confidence in operationalising dignity in care using a human rights‐based approach’ and results reveal dissonance between participants' beliefs about dignity and their ability to operationalise this value in practice. Dubiety emerged between what participants aspired to deliver and what they thought might be deliverable in a clinical setting in their capacity as students.

Most [> 90%] participants agreed that providing care with dignity is as much as about emotional empathy and agreed that nurses always had time to think about dignity whilst providing care [> 70%] and that they instinctively knew when they provided care with dignity [> 80%]. On the contrary, participants also agreed on how easy it was to lose sight of providing dignity in care when under pressure on placement, with > 50% participants being unsure if the current NHS culture was conducive to the provision of dignity in care.

Macaden et al. (2017) report nursing students' perceptions in relation to care of older adults from their pilot work on this research project. Participants in that study were confident in their ability to recognise practical aspects of dignity as it related to their professional code of conduct. They could identify promoters of patient dignity, again using language applied in their professional code of conduct. Participants could name environmental, organisational, personal and professional barriers to dignifying practice and the latter included ageism and discrimination towards older adults. They most significantly viewed dignifying practice as the patient being heard, having choices and involvement in decision‐making. Whilst participants in our study seemed to understand and conceptualise dignity within their practice context, their ability to operationalise dignity being inherently linked to one's human rights, is patently difficult for them.

The literature offers several perspectives that debate similar concerns over the (un)successful operationalisation of dignity in practice. Much of this literature is devoted to older patients and clients, particularly those with a diagnosis of dementia, but it is not limited to those groups. Dignifying care of patients with dementia is a source of frequent research and comment within the literature. Digby et al. (2017) found that people with dementia were considered a low priority and a ‘disruption to the normal routine’ (p. 1163). Management took priority over patient dignity, people with dementia were stigmatised, families were excluded from playing a role in care, nurses focused on tasks at the expense of specific patient's needs and support for nurses was found to be lacking (Digby et al. 2017). That review concluded that education and practical support, strong clinical leadership and role modelling were key requirements. A further review found that the over‐reliance on incontinence products is particularly evident in hospitals where ‘organisational sensitivity’ to individual needs is lacking. This establishes conditions within which the ‘loss of personhood and dehumanisation of people living with dementia can flourish’ (Boddington and Featherstone 2018, 258). These authors state that ward staff often feel helpless to improve care for people living with dementia, reporting that they lack the time, education and support to move away from an over‐reliance on products and deliver person‐centred care. Poor care is also said to reflect an excessive emphasis on safety at the expense of other patient needs (Scerri et al. 2020), and ‘unconscious institutional incompetence’. Some authors, such as Fekonja et al. (2022) offer a depressing indictment of the outcomes of undignified care upon older adults confined to bed. Fekonja et al. (2022) endorse Šanáková and Čáp's (2019, 10) terminology of ‘fractured dignity’ in these conditions. Participants clearly describe the losses associated with their confinement and the sub‐categories arising from Fekonja et al.'s (2022) phenomenological qualitative descriptive study include ‘dehumanising’ and ‘disrespectful’ care. Failure to maintain care directly impacts dignity as perceived by the participants. This is explained through loss of autonomy but also through the relationships with staff members that is, negative relationships were considered rude, leading to participants feeling inferior. Fekonja et al. (2022) conclude: ‘we found nursing care is mainly provided routinely, as the daily pre‐planned schedule of nursing activities like making beds, bathing, meals and changing underwear. When nursing care is provided routinely, under time pressure and staff shortages (Direckxe de Casterle et al. 2020) and with less compassionate care (Nathoo et al. 2021), the dignity of older people confined to bed is severely impaired’. (p. 2367).

Papastavrou et al. (2016) sought to explore how students perceived patients' dignity, seeking experiences with older adults and those with dementia in their narratives. Five themes to explain and categorise negative influences over patient dignity were developed in their analysis, and they offer an interesting, if disturbing, parallel with the data achieved in this study: (a) patients' preferences, verbal abuse and regarding a patient as a unique person; (b) privacy and confidentiality; (c) loss of autonomy and need for help; (d) discrimination; and (e) attribution and reciprocity. Verbal abuse (shouting at patients) was thankfully only reported in their study as tired staff being unintentionally short with patients, but the alignment of this terminology with ‘patient preferences’ and ‘recognising uniqueness’ might indicate it is also about the use of polite and respectful dialogue and about obtaining consent for any intervention. The participants in our study struggled to realise this aspect of nursing care, with questions about personalised aspects such as John's environment, or his hobbies and interests for example revealing limits to their perception of how they might honour John's uniqueness (Figure 3). Loss of autonomy and need for help was one of the categories arising in the Papastavrou et al. (2016) study, and students in our study scored disappointing levels of agreement where patients had diminished capacity. Ageism was manifest in the Papastavrou et al. (2016) findings. Privacy and confidentiality were alluded to in our study, where students understood that failing to pull the bed curtains fully and attempting to discuss very private matters where they could be overhead was not dignifying practice. Discrimination was reported in the Papastavrou et al. (2016) study as racism, and in fact, revealed students' personal vulnerability in the clinical learning environments. Attribution and reciprocity were evident within the free text answers participants gave for their proposed actions in response to undignified care, which included the role of reflection and the need to learn from such experiences. No free text responses alluded to increased satisfaction with the role of the registered practitioner, but it might be implicit in their unerring capacity to identify undignified care, even if they felt helpless to manage such situations at times.

A range of responses were uncovered when students were asked for their actions if they witnessed undignified care. Those who would speak to the member of staff directly were very much the minority. Many stated they would refer the incident to mentors or senior staff. Many more however believed it was ‘not their place’ as junior staff or simply that they did not feel sufficiently experienced to handle such conversations, believing they would be viewed as confrontational. Others worried about the consequences of ‘speaking‐up’ and used the incident to reflect on how they might deal with such events in the future. Papastavrou et al. (2016) recommend that student support services should be initiated for whistleblowing. No participant identified any role models from previous examples. There is significant evidence on undergraduate nursing students being expected to ‘police’ the quality of care in clinical practice, despite being reliant on clinical staff for their pastoral support, learning and assessment. The term initially applied was ‘don't rock the boat’ and this level of conformity and compliance by nursing students in clinical practice placements was a significant feature of that research (Levett‐Jones et al. 2009). Kyle et al.'s (2017) finding that dignity can be ‘unlearned’ becomes relevant here. This was a significant rationale for the new UK‐based Standards for Student Supervision and Assessment to support learning in the clinical environment whereby supervision and assessment were separated (NMC 2018). Bickhoff et al. (2016) identify moral courage as the moral value most closely aligned to students' likelihood of speaking‐up when undignified or poor care was witnessed. Bickhoff et al. (2016) identified four key themes arising from their qualitative descriptive research, about what motivated their participants to speak‐up, that is, (1) patient advocate identity, which had two sub‐themes of knowing one's own moral code and previous life experiences; (2) consequences to the patient and to the participant; (3) the impact of key individuals; and (4) picking your battles. This might suggest that this aspect of moral courage should be facilitated if a learning resource aimed to empower students within the clinical learning environment. There were participants in our study who used incidents of undignified care to endorse and confirm their patient advocate identity. Others were clearly articulating an internal debate between the consequences to the patient and to themselves. Mentors and senior staff appeared to be pivotal to ensuring dignity in practice if they were approachable. Further research could seek to understand any potential alignment between the frequency and intensity of undignified encounters with deployment of moral courage.

Informed by the findings from this survey, the research team has developed DigniSpace (2024), the first online interactive space for Dignity Education co‐produced with students focusing on the concept of dignity (through a consideration of human rights) that has been designed to help students learn more about the concept and to confidently promote and advocate dignity in practice.

This is the first such resource to empower students to interrogate the concept of Dignity using the Human Rights lens and become change agents to promote and advocate dignity in care as a fundamental human right in any practice context. Nursing students' vulnerability is more difficult to protect in an online space, and the use of self‐reflection will be promoted alongside the significance of human rights in supporting their interventions. This approach is viewed as necessary to positively influence cultures of care with a long‐term perspective. Perceptions and practices then develop, shift and are challenged as students shuttle between educational and real‐world experiences within the curriculum over time (Kyle et al. 2017). In this sense, findings and outputs from this research have used the context of nursing education as a critical opportunity by placing students at the heart of developing DigniSpace (2024) to support the sustainable development of a culture of confidence in delivering dignity in care.

### 5.1 Strengths and Limitations of the Work

The quantitative approach limits deduction arising from the data and indeed gaps in responses. The original study design included semi‐structured interviews, but this aspect was lost to the pandemic. Researchers have carefully constructed a discussion based upon the quantitative data, the free text responses within the questionnaire, the wider literature and the expertise of the multi‐disciplinary investigators. Further aspects of the human rights‐based approach and the co‐production aspects of this educational research will be presented in other paper/s.

## 6 Conclusions and Recommendations for Further Work

The results of the survey endorse undergraduate nursing students' beliefs that dignity in nursing practice is imperative. It reveals their lack of confidence to operationalise dignity in their professional practice. The results illuminate the implications of that deficit for patient care within their practice.

The participants talk about patients' dignity being undermined and nurses being able to respect dignity. Many students felt it was their own inherent sense of dignity that allowed them to sense or ask patients about their needs.

The study primarily aimed to explore students' understanding and conceptualisation of dignity using a human rights‐based approach and get them thinking about how they would operationalise dignity in care in a specific, hypothetical set of circumstances. Survey findings revealed participants' ambiguity or lack of agreement around dignity being associated with person‐centred care. There was a sense of students feeling disempowered or lacking confidence in responding to dignity breaches in care, whilst some participants felt equipped to challenge this by most usually referring on to clinical staff such as mentors and charge nurses due to the hierarchy in nursing systems within clinical contexts.

### 6.1 Implications for Nursing Education

Building on the ways in which student nurses intuitively understand dignity may be an effective means for developing ways to encourage dignity ‘learning’ at all levels of nursing training and practice. The focus on ways of learning from the perspective of individuals sits well with recognition of both the importance of practitioners being aware of the conceptual complexity of dignity (Jacobson 2012, 185) and of their responsibility as one crucial part of addressing gaps in dignity in care.

### 6.2 Impact

This paper reports findings on how undergraduate nursing students conceptualise and operationalise dignity. Students' responses illustrate confidence in conceptualising dignity and their limited confidence in how dignity can be practised in care contexts using a human rights‐based approach to care. The results of this study have been used to run co‐ – design workshops with students for the first time to develop an evidence‐based online Dignity education resource underpinned by the PANEL principles of the Scottish Human Rights Commission (Scottish Human Rights Commission 2024). The results also inform the wider debate regarding frameworks and models underpinning dignity in undergraduate nursing education and practice.

## 7 Reporting Method

The authors have adhered to the EQUATOR guidelines for reporting and have used A Consensus‐Based Checklist for Reporting of Survey Studies (CROSS).

## Author Contributions

L.M., E.W.: conceptualization. K.M.: data curation, formal analysis. L.M., E.W., L.E.: funding acquisition. L.M.: investigation. L.M., S.D., E.W., L.E.: methodology. L.M.: supervision. L.M.: project management. S.D., L.M.: writing – original draft. S.D., L.M., E.W., L.E.: writing – review and editing. All authors have read and agreed to this version of the manuscript.

## Conflicts of Interest

The authors declare no conflicts of interest.

## Data Availability

The data that support the findings of this study are available on request from the corresponding author. The data are not publicly available due to privacy or ethical restrictions.
